# Temperature–Stress Coupling Fatigue Behavior of Film-Cooling Holes in Complex Temperature Fields

**DOI:** 10.3390/ma17153785

**Published:** 2024-08-01

**Authors:** Dongxu Zhang, Zhenyu Xin, Zhuang Luo

**Affiliations:** College of Mechanical and Electrical Engineering, Shaanxi University of Science & Technology, Xi’an 710021, China

**Keywords:** nickel-based single-crystal superalloy, laser drilling, film-cooling holes, conjugate heat transfer, crystal plasticity finite element method

## Abstract

This research investigates the complex temperature distribution and fatigue behavior of single film-cooling holes manufactured by lasers with different pulse widths in a real flow field. The aerodynamic and heat transfer characteristics of film-cooling holes manufactured using lasers with different pulse widths were analyzed through laser drilling experiments, conjugate heat transfer simulations, and crystal plasticity finite element methods. The study investigated the relationship between changes in the geometric accuracy of the film-cooling holes and the corresponding flow and temperature fields during the film-cooling process. Additionally, the effects of temperature and structural variations on the stress around the holes in a flat plate composed of the second-generation nickel-based single-crystal superalloy DD6 in real flow and temperature fields were studied. The coupling effect of the temperature and stress fields around the holes on the fatigue behavior of the film-cooling holes was examined, and the fatigue damage mechanism of film-cooling holes in complex temperature fields was analyzed. It was found that changes in the blowing ratio do not affect the temperature and stress distributions around the holes but only alter the temperature peak. An increase in the temperature peak results in a decrease in the stress peak. Additionally, the fatigue damage of single film-cooling holes is determined by both the structural defects of the holes and the changes in material behavior due to the temperature around the holes, with the structural influence being more significant.

## 1. Introduction

In aero engines, turbine blades operate under extremely harsh service conditions, demanding exceptionally high reliability [1]. With the advancement of the aerospace industry and continuous innovations in engine technology, the most effective way to improve engine efficiency is to increase the turbine inlet temperature [2,3,4,5,6]. However, the current temperatures exceed the material limits of the turbine blades. Machining film-cooling holes on the blades has been proven to be the most effective cooling technique to date [7,8,9,10,11]. However, the presence of film-cooling holes reduces the structural continuity of the turbine blades, causing changes in the local stress–strain field around the holes. This leads to stress concentration, which can exacerbate blade fractures and other failure phenomena [12,13,14,15,16]. Therefore, machining high-quality film-cooling holes on turbine blades is crucial. Researchers have extensively studied the mechanical performance variations in film-cooling holes by employing different drilling techniques to alter their surface quality and geometric precision [17,18,19,20,21,22]. A. Bharatish et al. [23] conducted drilling experiments on alumina ceramic plates to study the effects of parameters such as the laser power, pulse frequency, scanning speed, and designed aperture on the quality of micro-holes. The experimental results revealed that the laser power and designed aperture significantly influenced the roundness of micro-holes, while the thermally affected zone and taper were affected by the laser pulse frequency. S. Döring et al. [24] conducted drilling experiments on silicon wafers using ultra-short pulse lasers to study the effects of processing parameters on the shape and depth of micro-holes. They found that the laser power determined the maximum depth of the micro-holes. However, due to the reduction in mechanical performance caused by the presence of film-cooling holes on blades, many researchers have further focused on the influence of micro-holes’ geometric surface quality on fatigue behavior. Chen [25] studied the influence of film-cooling hole roundness on its fatigue life using finite element analysis. The results showed that the smaller the roundness tolerance of the film-cooling holes, the longer the fatigue life. Li [26] investigated the fatigue crack initiation mechanisms and propagation modes using two drilling processes, electrical discharge machining (EDM) and laser drilling machining (LDM), from geometric, metallurgical, and mechanical perspectives using experimental and simulation methods. The study found that laser drilling resulted in a larger taper, lower roundness, and a thinner recast layer compared to electrical discharge machining (EDM). Ren et al. [27] conducted fatigue experiments at room temperature and utilized digital image correlation mapping and scanning electron microscopy (SEM) images to identify strain concentrations and predict crack initiation zones. Yin et al. [28] studied the tensile and compressive properties of anisotropic nickel-based single-crystal alloys using molecular dynamics simulations, explaining deformation processes from the perspective of dislocations.

However, the film-cooling process is a complex aerodynamic heat transfer phenomenon, which may generate intricate high-temperature fields inside the blades. Consequently, the fatigue fracture phenomena of film-cooling holes under high-temperature conditions differ significantly from those observed at room temperature. Hou et al. [29] developed a fatigue testing system with temperature gradients and conducted extensive fatigue experiments using alloy steel (30CrMnSi). The results indicate that high temperatures reduce the fatigue behavior of the materials. Furthermore, in subsequent work, Hou [30] established a fatigue model for nickel-based single crystals by considering crystallography and fatigue damage models under temperature gradients. The results also confirmed that temperature gradients significantly affect the fatigue behavior of nickel-based single crystals. Skamniotis et al. [31,32] studied the local stress issues between the holes and the base caused by thermal stresses in novel double-wall cooled turbine blades. By simplifying the complex temperature field during the film-cooling process to a uniform temperature field and using the analysis of maximum stress concentration factors, the study investigated the effects of the film-cooling hole tilt angle and ellipticity on the stress distribution. Modifications to global and local geometric features were made to enhance mechanical performance. However, under real operating conditions, the effect of film cooling on the blade temperature is not a simple constant temperature gradient. Many researchers [33,34,35] have conducted finite element simulations of the film-cooling process. The results indicate that different hole configurations lead to significant variations in the blade temperature field, and the temperature distribution in the solid domain is complex. While these studies have advanced the understanding of the film-cooling process, they often overlook the specific impacts of different drilling processes on the real temperature fields of film-cooling holes. Therefore, this research aims to fill this gap by analyzing the real temperature field changes around film-cooling holes produced by various drilling processes during the film-cooling process. Moreover, it is crucial to investigate how the geometric surface quality of film-cooling holes influences fatigue behavior in complex temperature fields and to understand the significance of different drilling processes in the fatigue damage mechanisms of these holes.

This study used laser drilling experiments with different pulse widths to establish realistic models of film-cooling holes. Based on conjugate heat transfer (CHT) simulations and crystal plasticity finite element method (CPFEM) fatigue simulations, it analyzed the aerodynamic and heat transfer characteristics of film-cooling holes manufactured by lasers with different pulse widths during the film-cooling process. The study explored the relationship between changes in the geometric accuracy of film-cooling holes and the corresponding flow and temperature fields. It investigated the coupled effects of temperature fields and stress fields around the holes under real flow-field temperature conditions on the fatigue behavior of film-cooling holes. Furthermore, this study analyzed the fatigue damage mechanism of film-cooling holes in complex temperature fields and established the relationship between the laser pulse width and the fatigue behavior of film-cooling holes.

## 2. Drilling Experiment and Real Hole Modeling

### 2.1. Experimental Materials and Drilling Process

The nickel-based single-crystal superalloy DD6 was chosen as the target material in this study, with its main elemental composition listed in Table 1. The specimens were flat plates with a thickness of 1 mm, and all samples were cut from cast rods with a specific crystal orientation of [001]. For the target alloy, the [001] orientation is the preferred growth direction, aligned along the z-axis of the crystal. This orientation was selected because it significantly enhances the material’s resistance to creep and deformation under high-temperature and high-stress conditions. The use of this orientation ensures the consistent directional properties of the material during experiments, thereby increasing the reliability of the results. The rods were sourced from experimental castings at the Aeronautical Materials Research Institute and underwent standard heat treatment (1290 °C for 1 h + 1300 °C for 2 h + 1315 °C for 4 h/AC + 1120 °C for 4 h/AC + 870 °C for 32 h/AC). The deviation of their crystal orientation from the [001] direction was within 10°. Through drilling experiments with different parameters, we found that the variations in laser drilling with different laser powers but the same pulse width were insignificant, while the differences between various pulse widths were substantial. Therefore, we selected several laser parameters for the experimental study. For nanosecond, picosecond, and femtosecond laser processing, only the laser power varied, while the laser frequency, feed rate, and duty cycle remained constant. Laser powers were set at 45 W, 14 W, and 2 W, respectively, with a frequency of 30 kHz, a single-layer feed rate of 0.05 mm/s, and a duty cycle of 90%.

**Figure 1:**
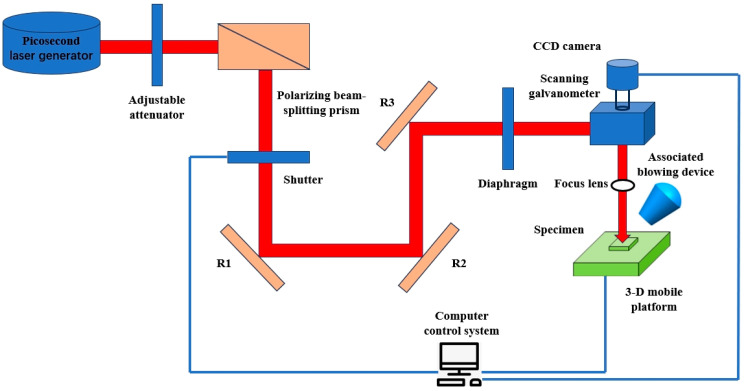
Schematic diagram of laser processing experiment system.

**Figure 2:**
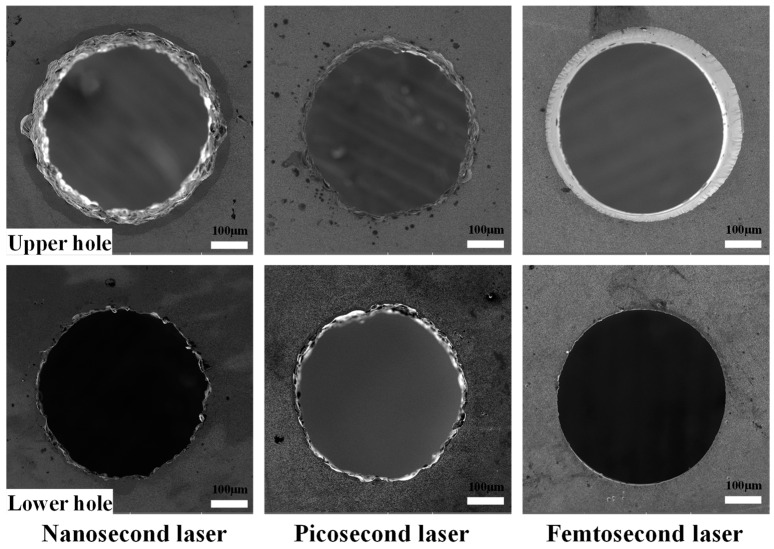
SEM images of film-cooling hole morphologies.

In the laser drilling experiment, the designed diameter D of the film-cooling holes was 0.4 mm. Three different pulse widths, namely, nanosecond, picosecond, and femtosecond lasers, were selected for the experiment. To control the experimental variables, only the laser power differed among the three laser processing techniques, while the laser frequency, duty cycle, and feed rate remained consistent. A schematic diagram of the laser processing system adopted in this work is shown in Figure 1. The surfaces of the film-cooling holes were observed using a scanning electron microscope (SEM), as shown in Figure 2. It can be seen that the film-cooling holes produced by the femtosecond laser process have smoother edges compared to the other two. The diameter and taper of the film-cooling holes also vary among the different laser processes. Therefore, it is essential to evaluate the geometric surface quality of the film-cooling holes produced by different laser pulse widths to study their impacts on the aerodynamic, heat transfer, and fatigue characteristics during the film-cooling process.

### 2.2. Geometric Accuracy Evaluation of Film-Cooling Holes

**Figure 3:**
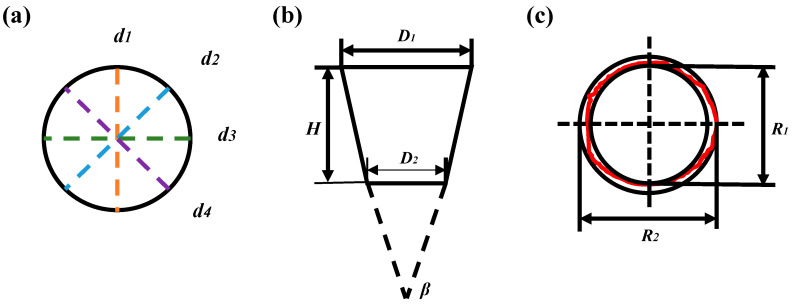
Measurement method for geometric accuracy of film-cooling holes. (**a**) Diameter; (**b**) conicity; (**c**) roundness.

The geometric accuracy of the film-cooling holes is primarily determined by their diameter, taper, and roundness. Figure 3 illustrates the measurement methods for assessing the geometric accuracy of the film-cooling holes.

The diameter of the hole was assessed using Image J (https://imagej.net/software/fiji/downloads, accessed on 29 July 2024) measurement software at eight equidistant points on both the upper and lower surfaces of the film-cooling hole. The average diameter was calculated using Equation (1):

(1) $$d=\frac{\left(d_{1}+d_{2}+d_{3}+d_{4}\right)}{4}$$

where $d_{1}$, $d_{2}$, $d_{3}$, and $d_{4}$ represent the actual diameters measured at the eight equidistant points of the film-cooling hole. The taper of the hole arises due to the prolonged exposure of the upper hole to the laser during the drilling process, leading to the excessive ablation of the substrate. Furthermore, debris accumulation inside the hole during continuous drilling may shield and hinder the laser beam, resulting in a taper where the upper hole diameter exceeds the lower hole diameter. The taper β can be determined using Equation (2):

(2) $$\beta =\tan^{-1}\left(\frac{D_{1}-D_{2}}{2H}\right)$$

where $D_{1}$ and $D_{2}$ represent the upper and lower hole diameters of the film-cooling hole, respectively, and H stands for the plate thickness. Moreover, roundness is another crucial factor affecting the geometric accuracy of the film-cooling hole. The roundness error is defined as the difference in radii between two concentric circles, one being the minimum inscribed circle of the film-cooling hole $R_{1}$ and the other being the maximum inscribed circle $R_{2}$. The roundness error value indicates how closely the hole profile resembles an ideal circle. A smaller roundness error suggests that the film-cooling hole contour approaches an ideal circle more closely. The roundness error, denoted by r, is calculated using Equation (3):

(3) $$r=\frac{\left(R_{1}-R_{2}\right)}{2}$$

The geometric surface quality parameters of the film-cooling holes fabricated by different pulse widths are listed in Table 2. It can be found that the geometric surface quality of the film-cooling holes is significantly affected by the laser pulse width, and the hole diameter error of case 2 reaches a maximum of 34.8% compared to case 1; in terms of the roundness error, the errors of cases 2 and 3 are also significantly higher than those of cases 1 and 4, whereas the taper of case 3 has the best performance among the three processed holes formed by the three hole-making processes.

### 2.3. Real Geometric Modeling of Film-Cooling Holes

**Figure 4:**
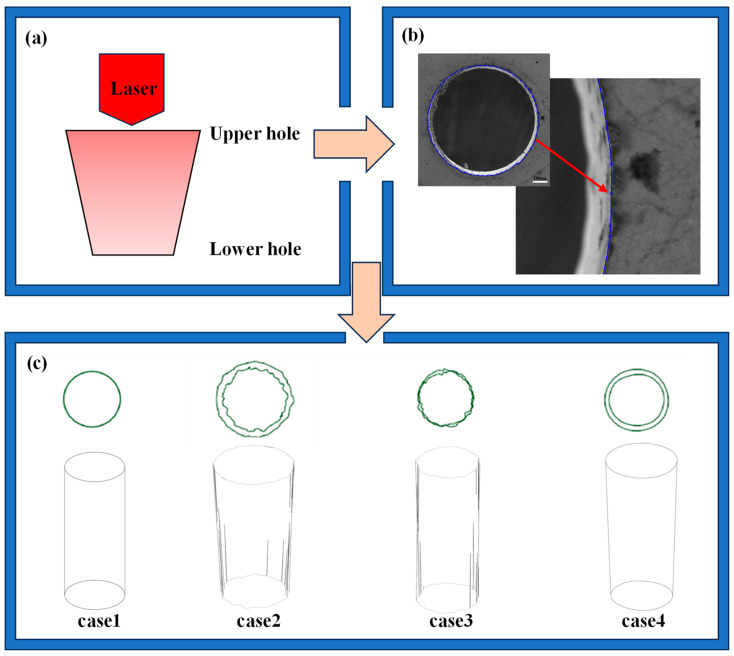
The geometric model for the formation of film-cooling holes: (**a**) hole contour tracing points; (**b**) a schematic diagram of laser drilling; (**c**) real CAD hole edges and 3D models.

Based on the evaluation of the geometric accuracy of film-cooling holes made by lasers with different pulse widths, as described earlier, four hole types, as listed in Table 2, were chosen. Using SolidWorks (2018) 3D software and actual SEM images of film-cooling holes, geometric models of these holes produced by lasers with various pulse widths were created. By marking points along the profiles of both the upper and lower holes of the film-cooling hole, the actual profiles were established, excluding the thermally affected region. Subsequently, a three-dimensional model of the actual film-cooling hole was developed, as depicted in Figure 4.

## 3. Nickel-Based Single-Crystal Fatigue Constitutive Model

### 3.1. Fatigue Constitutive Model

The crystal slip model was first proposed and derived by Talor et al. [36]. Expanding on this theoretical groundwork, Hill and Rice et al. [37] detailed the geometric and kinematic aspects of crystal plastic deformation. Later, Asaro and Peirce et al. [38] extensively discussed the constitutive response of crystal plasticity, offering crucial insights into the behavior of crystal plasticity constitutive models. The fatigue simulations in this study are based on the crystal plasticity constitutive model introduced in references [39,40], incorporating rate-dependent constitutive relations and kinematic hardening criteria. This model assumes that the shear stresses of each slip system are connected to the macroscopic stress and are defined as

(4) $$\tau^{\left(\alpha \right)}=\sigma :P^{\left(\alpha \right)}$$

where $\tau^{\left(\alpha \right)}$ represents the shear strain in slip system α, σ denotes the stress tensor in the crystallographic coordinate system, and P^(*α*)^ represents the orientation factor in slip system α, defined as

(5) $$P^{\left(\alpha \right)}=\frac{1}{2}\left(m^{\left(\alpha \right)}n^{{\left(\alpha \right)}^{T}}+n^{\left(\alpha \right)}m^{{\left(\alpha \right)}^{T}}\right)$$

where m^(*α*)^ represents the slip direction in slip system α in the initial configuration, and n^(*α*)^ represents the unit normal vector to the slip plane in slip system α in the initial configuration.

The shear strain rate ${\dot{\gamma }}^{\left(\alpha \right)}$ and shear stress $\tau^{\left(\alpha \right)}$ on slip system α are described by the following power-law relationship:

(6) $${\dot{\gamma }}^{\left(\alpha \right)}=\mathrm{sgn}\left(\tau^{\left(\alpha \right)}-X\right){\dot{\gamma }}_{0}^{\left(\alpha \right)}{\left[\left|\frac{\tau^{\left(\alpha \right)}-X}{g^{\left(\alpha \right)}}\right|\right]}^{\frac{1}{m}}$$

where ${\dot{\gamma }}^{\left(\alpha \right)}$ is the initial shear strain rate, $g^{\left(\alpha \right)}$ is the reference shear stress, and m is the strain rate sensitivity exponent. When selecting a small value for the strain rate sensitivity exponent, the material response becomes indifferent to the actual rate. In the equation, $\mathrm{sgn}\left(\right)$ denotes the sign function, where $\mathrm{sgn}\left(x\right)=-1$ when x<0 and $\mathrm{sgn}\left(x\right)=1$ when x≥0. In this context, m=0.02 and ${\dot{\gamma }}_{0}^{\left(\alpha \right)}=0.001$.

For the low-cycle fatigue of single-crystal superalloys, the back stress *X* is characterized using a kinematic hardening criterion. Building upon the kinematic hardening criterion introduced by Yaguchi et al. [41], a kinematic hardening rule tailored for low-cycle fatigue is proposed, with its evolution equation formulated as follows:

(7) $$\dot{X}=C\left\{a{\dot{\gamma }}^{\left(\alpha \right)}-\left(X-Y\left|{\dot{\gamma }}^{\left(\alpha \right)}\right|\right)\right\}-d\exp\left(\frac{-Q}{RT}\right){\left|X\right|}^{n}\mathrm{sgn}\left(X\right)$$

(8) $$\dot{Y}=-a\left\{Y_{st}\mathrm{sgn}\theta \left(X\right)+Y\right\}{\left|X\right|}^{n}$$

where C, a, d, Q, R, α, and n are material constants for the kinematic hardening law of nickel-based single crystals, as shown in Table 3. T represents the absolute temperature, Y represents the dynamic recovery term of the back stress, and $Y_{st}$ is the extremum of Y.

The strain hardening of the material evolves as a function of ${\dot{g}}^{\left(\alpha \right)}$:

(9) $${\dot{g}}^{\left(\alpha \right)}=\sum_{\beta }h_{\alpha \beta }\left|{\dot{\gamma }}^{\left(\beta \right)}\right|$$

where $h_{\alpha \beta }$ is the hardening coefficient, which determines the hardening of slip system β by the slip shear amount in slip system α, and it satisfies

(10) $$h_{\alpha \beta }=q_{\alpha \beta }h_{\beta }$$

(11) $$h_{\beta }=h_{0}{\left(1-\frac{g_{\alpha }}{\tau_{s}}\right)}^{\beta }$$

where $q_{\alpha \beta }$ is the extremum of the latent hardening ratio parameter, $h_{\beta }$ is the single hardening rate, $h_{0}$ is the hardening modulus, and $\tau_{s}$ and β are model parameters satisfying $\tau_{s}/\tau_{0}=1.5$, $h_{0}/\tau_{0}=1.2$, and β=1.3.

The low-cycle fatigue damage model used in this study was proposed by Tinga [42], who considered only the fatigue behavior of nickel-based superalloys in the [001] orientation by activating octahedral slip systems with the Schmid factor $S_{f}=0.4082$. Hou et al. [30] reduced the number of variables by expressing $S_{oct}$ as a function of $\tau_{0}$ and $m_{oct}$ as a function of t, defining fatigue $\Delta D_{i}^{fat}$ damage as

(12) $$\Delta D_{i}^{fat}=\sum_{\alpha =1}^{12}{\left[\frac{\left|\tau^{\alpha }\right|}{2.5\times \tau_{0}}\right]}^{\frac{t}{100}}{\left[\frac{{\dot{\gamma }}_{\max}^{\alpha }}{10}\right]}^{n_{fat}}\exp\left(\frac{-Q^{C}}{RT}\right)$$

where t is the Celsius temperature, T is the absolute temperature, R and $Q^{C}$ are model parameters, and $n_{fat}$ is the fatigue damage index.

In this fatigue constitutive model, the material parameters vary with temperature and are derived experimentally. Table 4 outlines the fatigue damage model parameters for the nickel-based superalloy DD6 across different temperature ranges. They are specifically determined as follows:

(1) Assuming that C, a, d, $Y_{st}$, and $n_{fat}$ are all equal to zero, Young’s modulus *E*, Poisson’s ratio *v*, the shear modulus *G*, $\tau_{0}$, and $g_{0}^{\alpha }$ are determined from the stress–strain curves at different temperatures. For nickel-based single-crystal DD6, $\tau_{0}$ and $g_{0}^{\alpha }$ are highly temperature sensitive. Thus, they are calculated using the following equation:
  (13) $$\tau_{0}=\tau_{0,T_{1}}+\frac{\tau_{0,T_{2}}-\tau_{0,T_{1}}}{T_{2}-T_{1}}\left(T-T_{1}\right)$$
  (14) $$g_{0}^{\alpha }=g_{0,T_{1}}^{\alpha }+\frac{g_{0,T_{2}}^{\alpha }-g_{0,T_{1}}^{\alpha }}{T_{2}-T_{1}}\left(T-T_{1}\right)$$
(2) Through experiments, the trial-and-error method is used to determine C, a, d, Q, R, α, n, and $Y_{st}$, as shown in Table 3.
(3) For $n_{fat}$, obtained by fitting through low-cycle fatigue experiments..

### 3.2. Validation of Constitutive Models

**Figure 5:**
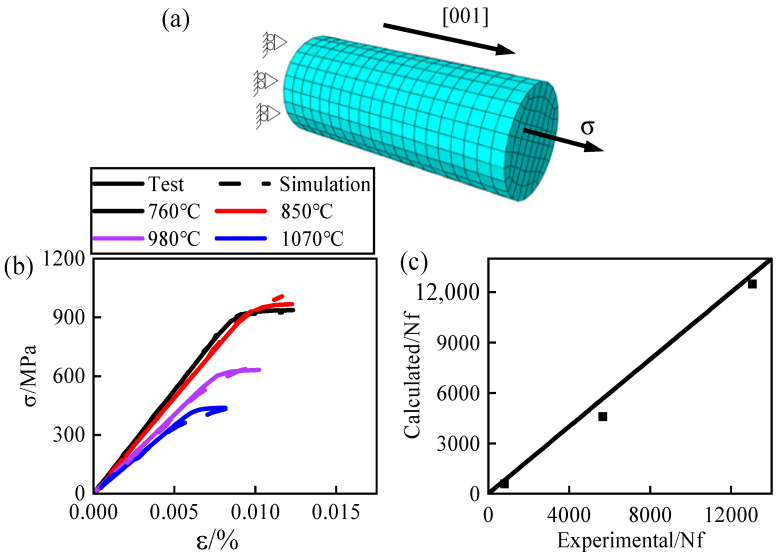
Verification of the stress-damage model: (**a**) finite element model and boundary conditions of the fixed-diameter section; (**b**) stress–strain curves at different temperatures; (**c**) experimental and predicted lifespan at 760 °C.

The crystal plasticity fatigue constitutive model described earlier utilizes temperature interpolation to simulate continuous temperature ranges. This was achieved by developing a UMAT user subroutine and integrating it into ABAQUS for calculations. To verify the accuracy of this subroutine, numerical simulations were conducted based on experimental data sourced from the *Chinese Superalloys Handbook* [43]. The agreement between experimental and simulated results was then assessed. Using the material handbook, a finite element model was created, as depicted in Figure 5a, with the tensile direction set to [001]. Tensile simulations were performed at four different temperatures: 760 °C, 850 °C, 980 °C, and 1070 °C. Additionally, low-cycle fatigue simulations were executed at 760 °C. The comparison between experimental and simulated results at different temperatures shows favorable agreement, as illustrated in Figure 5b,c. This demonstrates the applicability of the stress-damage model employed in this study for analyzing the low-cycle fatigue life of nickel-based single-crystal DD6 across varying temperatures.

### 3.3. Numerical Simulation Methods and Models

#### 3.3.1. Domain Model and Boundary Conditions

In this study, conjugate heat transfer (CHT) numerical simulations were conducted using the commercial software ANSYS CFX 2020 (https://www.ansys.com/products/fluids/ansys-cfx, accessed on 29 July 2024). The simulations employed implicit coupling and decomposition techniques. The turbulence model utilized was the k−ω SST (Shear Stress Transport) model with the Gamma–Theta transition model. Second-order upwind schemes were used for all parameters to enhance computational accuracy. Convergence criteria were set to achieve residuals below 10^−5^ for all flow parameters, with the solid domain maintaining constant temperatures around the film-cooling holes.

**Figure 6:**
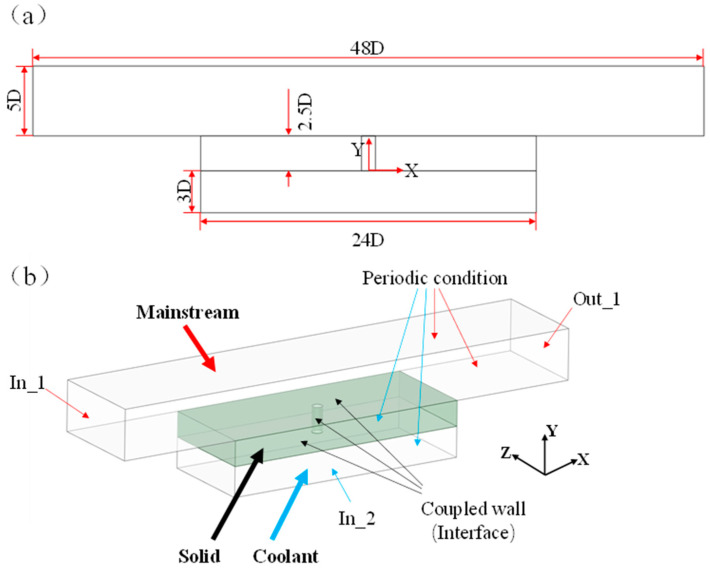
Single-hole CHT numerical simulation model: (**a**) calculation of area size, (**b**) boundary conditions.

The computational domain includes the mainstream fluid region, the coolant fluid region, and the solid region. All computational domains are symmetric with respect to the XY plane and YZ plane. Only the morphology of the film-cooling holes is varied, as shown in Figure 4c. Figure 6a presents the dimensions of the entire computational domain for the conjugate heat transfer (CHT) numerical simulation. The dimensions of the mainstream fluid region are 10 D × 48 D × 5 D (width × length × height), the coolant fluid region measures 10 D × 24 D × 3 D, and the solid region measures 10 D × 24 D × 2.5 D (width × length × height). Figure 6b illustrates the boundary conditions used in the CHT numerical simulation. In the mainstream fluid region, X/D = −24 represents the inlet boundary for the mainstream flow, using a velocity inlet condition with a temperature of 1300 K and a velocity of 150 m/s in the positive X-direction. X/D = 24 represents the mixed-gas outlet boundary, employing a pressure outlet condition set to standard atmospheric pressure. In the coolant fluid region, Y/D = −3 represents the inlet boundary for the coolant flow, utilizing a velocity inlet condition with a temperature of 650 K. The velocity is in the positive Y-direction and is determined by the blowing ratio M. In this study, the turbulence intensity for both the mainstream and coolant flows is set to 5%. The blowing ratio *M* is configured as 0.3, 0.6, and 0.9, representing low, medium, and high blowing ratios, respectively. These settings simulate aerodynamic and heat transfer phenomena during film-cooling processes under different blowing ratios. The blowing ratio *M* is defined as

(15) $$M=\frac{\rho_{c}u_{c}}{\rho_{m}u_{m}}$$

where $\rho_{c}$ is the density of the coolant flow (kg/m³), $\rho_{m}$ is the density of the mainstream flow (kg/m³), $u_{c}$ is the velocity of the coolant flow at the film-cooling hole exit (m/s), and $u_{m}$ is the velocity of the mainstream flow (m/s). Both the mainstream and coolant flows have a turbulence intensity of 5%. The blowing ratio *M* is set to 0.3, 0.6, and 0.9, respectively, to simulate aerodynamic and heat transfer phenomena during film cooling at low, medium, and high blowing ratios. The sidewalls at Z/D = 2 and Z/D = −2 employ periodic boundary conditions to simulate the flow and heat transfer of an infinite array of film-cooling holes on the blade. At the interface between the fluid and solid regions and on the inner surface of the film-cooling holes, conjugate heat transfer conditions are applied. In the CHT analysis, the temperature and heat exchange at the coupling interfaces between the fluid and solid regions satisfy [44]

(16) $$T_{m}=T_{c}$$

(17) $$-k_{c}\frac{\partial T_{c}}{\partial n}=k_{m}\frac{\partial T_{m}}{\partial n}$$

where $T_{ci}and{\;T}_{mi}$ represent the temperatures (K) on the coolant side and mainstream side, respectively. $k_{c}$ and $k_{m}$ are the thermal conductivity coefficients of the coolant and mainstream regions, respectively, and n is the unit normal vector to the coupling interface. All other surfaces are set as smooth, non-slip adiabatic walls.

Additionally, due to the significant temperature difference between the mainstream and coolant flows and the wide temperature range involved in the perforated plate, the thermal parameters of the nickel-based single-crystal high-temperature alloy DD6 are considered temperature-dependent according to the *Chinese High-Temperature Alloy Handbook* [43], as shown in Table 5. Furthermore, within the engine, which operates in high-temperature environments, the impact of thermal radiation is minimal, and therefore, it is neglected in the CHT numerical simulations in this study [45,46]. However, the CPFEM fatigue simulation model is relatively simple, and its computational domain model and boundary conditions are discussed together during mesh partitioning.

#### 3.3.2. Mesh Partitioning and Mesh Independence Validation

**Figure 7:**
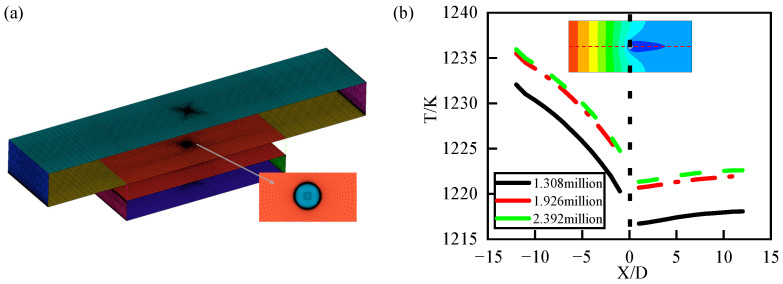
(**a**) Mesh division of single-hole CHT model. (**b**) Verification of grid independence in single-hole CHT model.

This study used ICEM CFD software (https://innovationspace.ansys.com/forum/forums/topic/icem-download-installation/, accessed on 29 July 2024) to perform hexahedral structured mesh partitioning of the computational domain model for the CHT simulation of individual film-cooling holes, discretizing the computational region. To enhance mesh accuracy and improve computational convergence, O-type structured mesh partitioning was employed at the film-cooling hole locations, maintaining an overall mesh quality above 0.6, as shown in Figure 7a. Based on the choice of turbulence model, using the k−ω SST turbulence model with the Gamma–Theta transition model requires the near-wall mesh to resolve the viscous sublayer with y^+^ < 1. Therefore, the first-layer height of the mesh is set to 1 × 10^−7^ m, with a growth rate of 1.1. Based on this, grid refinement analysis was conducted to validate grid independence. As shown in Figure 7b, temperatures at the characteristic line (Z = 0) were compared among three different grid densities (1.308 million, 1.926 million, and 2.392 million).

In Figure 7b, it can be observed that as the number of grid cells increases, the overall cooling efficiency along the characteristic line (Z = 0) remains consistent, but the overall temperature values change accordingly. When the number of grid cells increased from 1.926 million to 2.392 million, the overall cooling efficiency showed minimal variation, gradually approaching a stable value. To balance the prediction accuracy and computational cost, a grid density of around 1.926 million cells was chosen for subsequent CHT simulations.

**Figure 8:**
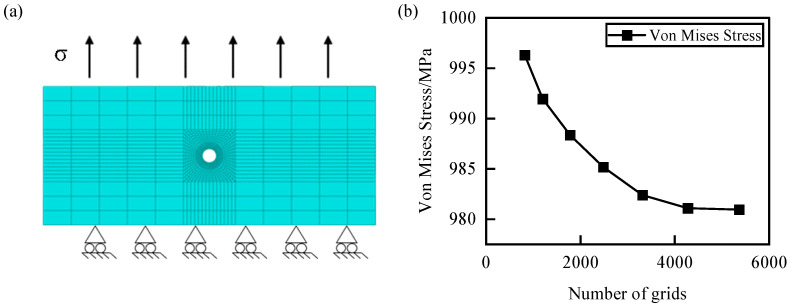
Verification of grid independence in single-hole CPFEM model. (**a**) Boundary condition; (**b**) Grid independence verification.

Figure 8a illustrates the finite element model and boundary conditions for the CPFEM fatigue simulation using eight-node hexahedral elements (C3D8) in ABAQUS 2020. An O-grid mesh partitioning strategy was employed at the gas film holes to enhance mesh quality and model convergence. The temperature was simulated as an isothermal field or the complex temperature field obtained from the aforementioned CHT simulation. The loading stress was controlled to maintain a minimum cross-sectional stress level of 540 MPa along the positive Z-axis direction, with a loading frequency of 3 Hz and a stress ratio R of 0.1. Additionally, grid independence validation was conducted by controlling the number of grid nodes around the holes for mesh refinement, as shown in Figure 8b. It can be observed that as the number of grid cells increases, the stress values gradually decrease. When the number of grid elements reaches 4272, the stress values at the same point around the hole stabilize. Therefore, the final number of elements for the fatigue simulation model of a single gas film hole is determined to be 4272.

## 4. Conjugate Heat Transfer Analysis

### 4.1. Analysis of Overall Cooling Effect of Film-Cooling Holes

**Figure 9:**
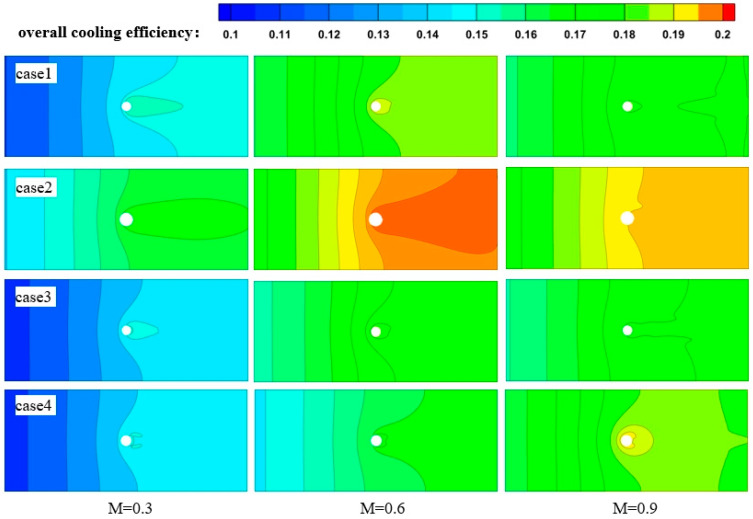
Overall cooling efficiency distribution on the wall surface (Z = 1).

Figure 9 illustrates the distribution of the overall cooling effects of laser-manufactured film-cooling holes with different pulse widths on the mainstream side (Z = 1) wall surface at various blowing ratios (M = 0.3, 0.6, 0.9). The overall cooling effect T is obtained from Equation (18). It can be observed that with any hole configuration and blowing ratio, the overall cooling effect downstream of the film-cooling holes is greater than that upstream, and the cooling effect distribution upstream of the holes is more uniform. This is because downstream of the film-cooling holes, the overall cooling effect results from both impingement cooling and film cooling acting together, whereas upstream of the holes, only impingement cooling exists, and convective heat transfer inside the holes is limited to the vicinity of the holes.

(18) $$\phi =\frac{T_{m}-T}{T_{m}-T_{c}}$$

where $T_{m}$ is the mainstream temperature, $T_{c}$ is the cold flow temperature, and T is the mainstream-side coupling wall temperature.

For the cooling effect upstream of the holes, at the same blowing ratio, the diameter of film-cooling holes varies due to different drilling processes. When the downstream hole diameter increases, the mass flow rate of cooling air increases. As a result, film-cooling holes with larger diameters, such as those from nanosecond drilling (case 2), exhibit relatively optimal cooling effects. However, for the cooling effect downstream of the holes, at low blowing ratios (M = 0.3), the presence of film cooling can be clearly observed. This results in uneven cooling regions distinct from those upstream of the holes. As the blowing ratio increases, the degree of uneven cooling gradually diminishes. This is because as the blowing ratio increases, the momentum of the cooling jet increases, leading to higher lift-off heights and increased penetration into the mainstream, which weakens the effectiveness of film cooling. However, for nanosecond drilling (case 2) and femtosecond drilling (case 4), due to their larger upstream hole diameters, the jet velocity upon exiting the holes decreases. As a result, significant film-cooling phenomena are still observed even at high blowing ratios (M = 0.9).

**Figure 10:**
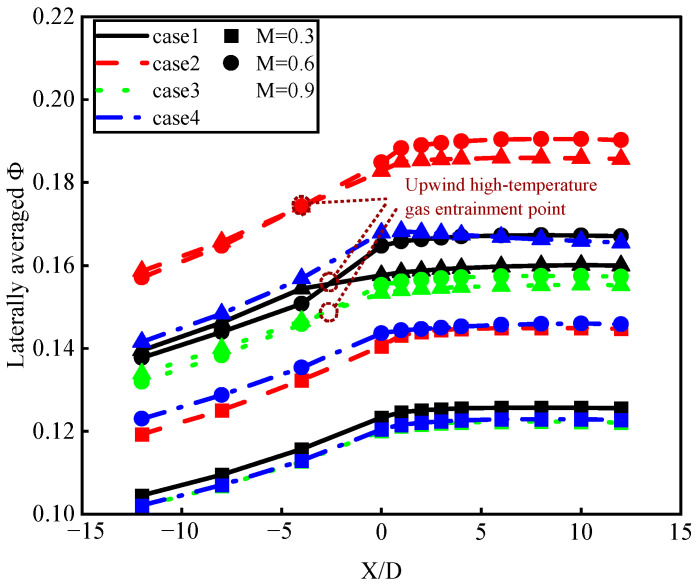
Horizontal average overall cooling efficiency flow distribution.

Figure 10 illustrates the axial distribution of the lateral average overall cooling efficiency of laser-manufactured film-cooling holes with different pulse widths at various blowing ratios (M = 0.3, 0.6, 0.9). It can be observed that with different hole configurations and blowing ratios, the axial trend of increasing lateral average overall cooling efficiency remains consistent. This is because the cooling mechanism at the same position in the film-cooling process is uniform. Among the three cooling methods, the impingement cooling effect remains constant throughout. The closer the distance from upstream of the hole (−12 < X/D < 0) to the hole, the greater the extent of convective heat transfer inside the hole, showing a significant increasing trend. Downstream of the hole (0 < X/D < 5), the addition of film cooling further enhances the cooling effect, reaching its maximum value. Downstream of the hole (5 < X/D < 12), the film-cooling effect weakens due to insufficient momentum of the cooling jet at the far end and an increase in film temperature caused by the mixing of coolant and mainstream flows. As a result, impingement cooling predominates, leading to a relatively constant lateral average overall cooling efficiency. Toward the far end downstream of the hole, where the cooling jet dissipates completely, there is a slight downward trend in cooling efficiency.

Combined with the three cooling methods generated during the air film-cooling process, the above phenomenon is due to the variation in the aperture diameter of the film-cooling holes manufactured by different perforation processes, which, in turn, affects the cooling method during the air film-cooling process. In the region where −12 < X/D < −2.5, impingement cooling predominates. As the blowing ratio increases, the mass flow rate of the cooling air increases, thereby enhancing the effectiveness of impingement cooling. At X/D = −2.5, the variation in the hole diameter affects the airflow vortex inside the hole, leading to changes in convective heat transfer effectiveness within the hole. In the region downstream of the hole where 0 < X/D < 12, the variation in the hole diameter affects the velocity of the cooling jet exiting the hole. At a high blowing ratio (M = 0.9), this results in the increased lift-off height of the cooling jet, which weakens the film-cooling effect. The enhanced impingement cooling effect due to the higher blowing ratio cannot fully compensate for the reduced film-cooling effect. Consequently, this leads to a situation where the cooling effectiveness at a moderate blowing ratio (M = 0.6) surpasses that at a high blowing ratio (M = 0.9).

Based on the overall cooling effect distribution (Figure 9), it can be observed that at the same blowing ratio, there is minimal difference in cooling effectiveness between the ideal hole (case 1) and picosecond drilling (case 3). The ideal hole (case 1) shows slightly superior cooling effectiveness across various blowing ratios. This is because the slightly smaller diameter of holes formed by picosecond drilling (case 3) results in a minor fluctuation in cooling effectiveness. Therefore, the roundness error caused by different drilling processes has no significant impact on the aerodynamic and heat transfer characteristics of film cooling.

### 4.2. Behavior of Temperature Field of Film-Cooling Holes

**Figure 11:**
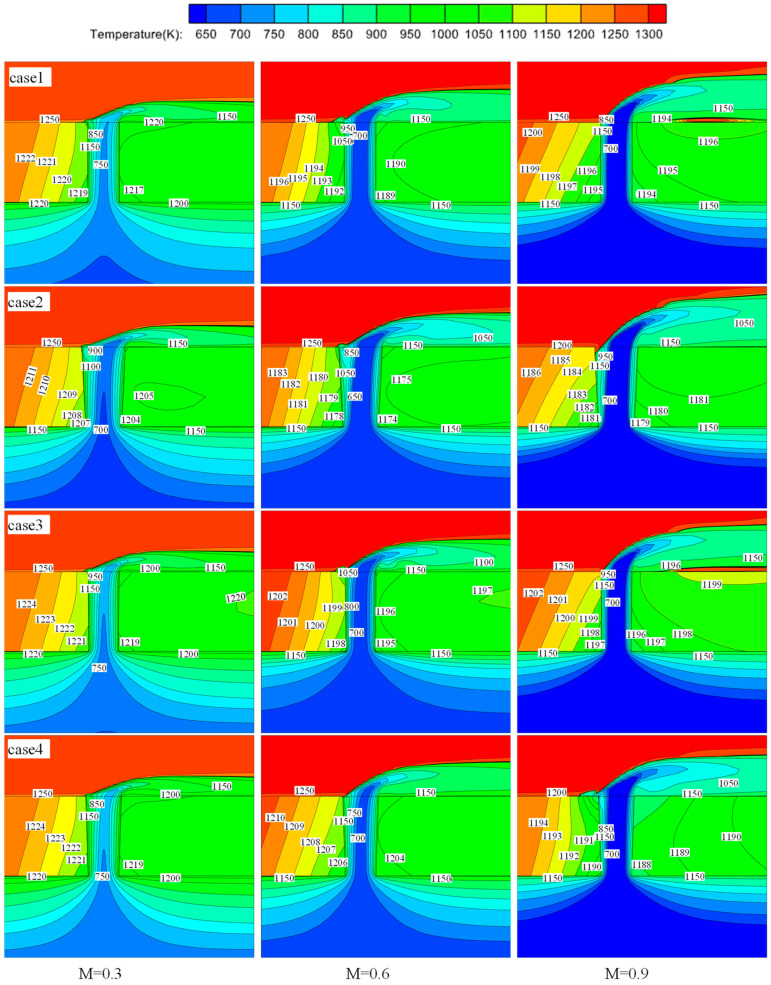
Plane (Y = 0) temperature distribution.

Figure 11 shows the temperature distribution at the plane (Y = 0) at various blowing ratios (M = 0.3, 0.6, 0.9). It can be observed that with increasing blowing ratio or hole diameter, the coolant temperature entering the hole decreases, leading to enhanced heat transfer inside the hole. This is due to the increased mass flow rate of the coolant. However, as the lift-off height of the cooling jet increases, the effectiveness of film cooling decreases. This effect is particularly pronounced for the ideal hole (case 1) and picosecond drilling (case 3), where the smaller upstream hole diameter causes a significant rise in the film at a high blowing ratio (M = 0.9), leading to localized high temperatures in the solid region downstream of the hole without film coverage.

Comparing the temperature field distributions in the solid domain, it is observed that at low to moderate blowing ratios (M = 0.3, 0.6), the temperature distributions on perforated flat plates manufactured using different drilling processes are similar. This is because, at lower blowing ratios, variations in hole configurations have a less significant impact on the cooling mechanism but rather affect the magnitude of cooling effectiveness. However, at a high blowing ratio (M = 0.9), variations in the hole diameter significantly influence the composition of the cooling mechanism. Among them, the temperature field distributions on the solid domain plane (Y = 0) upstream of the holes for the ideal hole, nanosecond drilling, and picosecond drilling (cases 1–3) are similar. In contrast, femtosecond drilling (case 4) exhibits a temperature field distribution similar to those observed at lower blowing ratios (M = 0.3, 0.6) for the aforementioned three cases.

**Figure 12:**
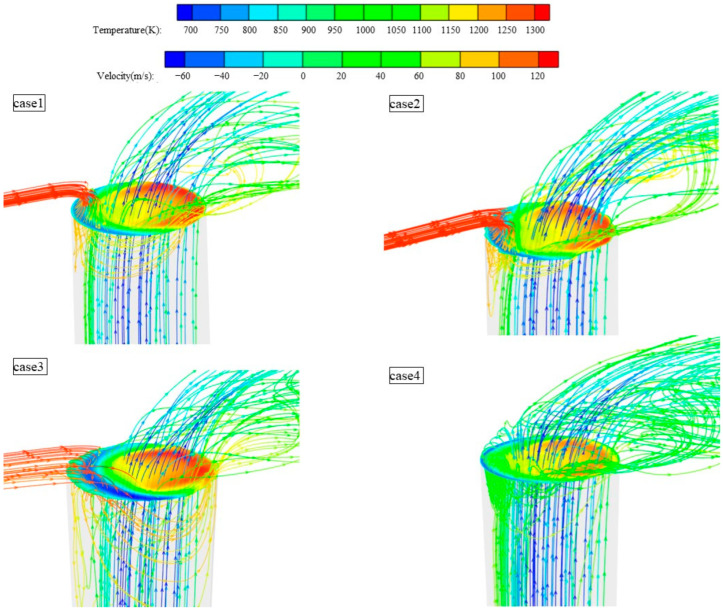
Eddy currents inside different hole types at M = 0.9.

The phenomenon described is due to the variation in the hole diameter affecting vortex formation inside the holes for the ideal hole, nanosecond drilling, and picosecond drilling (cases 1–3) at high blowing ratios. This variation consequently impacts convective heat transfer upstream of the holes. Figure 12 illustrates the vortex distribution inside different types of holes at a blowing ratio of M = 0.9. The contour plot at the outlet shows the velocity magnitude of the jets along the Y-axis, while streamlines depict the temperature and flow direction of the mainstream and jets. It can be observed that when the cooling jet exits the hole with higher momentum, on the windward side (where the hot flow impacts the cold jet), the cooling jet and mainstream flow interact by shearing, leading to more entrainment of the mixed airflow into the hole. In cases 1 to 3, significant negative velocity gradients along the Y-axis are observed on the windward side for ideal, nanosecond, and picosecond laser drilling. The mainstream high-temperature fluid enters the gas film hole, where its temperature is higher than that of the cooling jet’s mixed flow. This part of the flow weakens the cooling effect of the flow–solid heat transfer near the windward solid domain. In contrast, for picosecond laser drilling (case 4), there is no involvement of mainstream high-temperature gas, only a small part of the cooling jet producing vortex recirculation. This explains why, in the lateral average overall cooling efficiency distribution (Figure 10), ideal, nanosecond, and picosecond drilling (cases 1–3) intersect at blowing ratios M = 0.6 and M = 0.9.

For the temperature distribution in the solid domain plane (Y = 0), as shown in Figure 10, multiple-order thermal distribution characteristics of the temperature field for a single-hole plate are proposed. The first-order thermal characteristics are controlled by film cooling, manifested in the temperature gradient distribution along the thickness direction of the hot wall surface downstream of the hole. The second-order thermal characteristics are governed by heat transfer between the gas and solid, manifested in the cooling of the solid near the cold flow side and around the hole by the low-temperature gas. This is evidenced by the lowest temperatures around the hole perimeter and the outward expansion of temperature gradients with increasing temperatures.

The temperature values in the solid domain are correlated with the extent of different cooling methods during film cooling. At low to moderate blowing ratios (M = 0.3, 0.6), the cooling methods of film-cooling holes manufactured by various perforation techniques exhibit consistent behaviors. They all involve first-order thermal characteristics related to film cooling, as well as second-order thermal characteristics such as impingement cooling and gas–solid heat exchange within the hole. At a high blowing ratio (M = 0.9), the cooling mechanisms change due to the lifting of the cooling jet and the involvement of mixed gases on the windward side in the holes. Comparing the temperature values around the holes, it is found that nanosecond laser drilling (case 2) consistently maintains the lowest temperatures. This is because the larger manufacturing errors in nanosecond laser processing (case 2) result in larger hole diameters compared to the design dimensions, which enhances the effectiveness of impingement cooling and film cooling. This maintains optimal cooling performance even with the involvement of mixed gases.

## 5. Fatigue Damage Analysis

### 5.1. Fatigue Behavior of Film-Cooling Hole Structures in Isothermal Fields

Fatigue damage or fatigue behavior is influenced by both the temperature field around the holes and geometric accuracy [25,30]. To clearly analyze the influence of different factors, a fatigue damage analysis was performed on film-cooling holes with varying geometric accuracies in a uniform temperature field. To investigate the impact of the aperture, roundness, and taper on the fatigue behavior of film-cooling holes, simulations were conducted using different hole-manufacturing processes: ideal holes (case 1), holes formed by picosecond drilling (case 3), and tapered holes (case 5). The geometric accuracy parameters of the film-cooling holes are detailed in Table 6.

**Figure 13:**
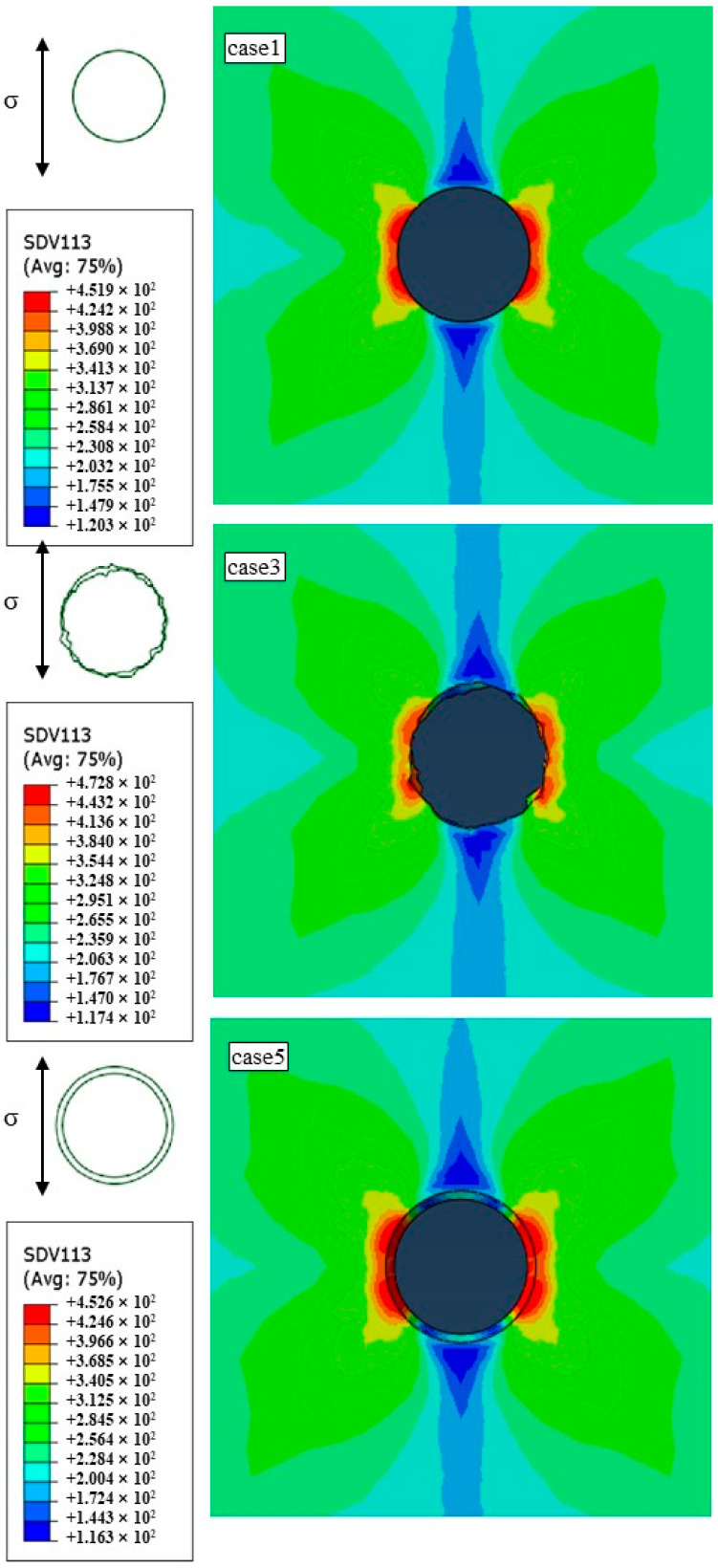
Cloud map of maximum resolve shear stress in gas film-cooling holes in 900 °C isothermal field.

The distribution of the maximum resolve shear stress around the hole perimeter determines the initiation of slip during the fatigue process of nickel-based single crystals. Numerous studies [25,30,47,48,49] have demonstrated that the maximum resolve shear stress based on crystal plasticity theory can effectively predict fatigue damage paths and plastic strain regions. Figure 13 shows the contour maps of the maximum resolve shear stress for film-cooling holes with different geometric accuracies under isothermal conditions at 900 °C. It can be observed that variations in the hole diameter, taper, and roundness do not significantly affect the distribution of the maximum resolve shear stress around the hole perimeter. The stress distribution exhibits a butterfly-shaped pattern, with low-stress regions along the loading direction and noticeable stress gradients perpendicular to the loading direction. However, compared to the ideal hole (case 1) and tapered hole (case 5), picosecond drilling (case 3) with a larger roundness error shows a discontinuous distribution of the maximum resolve shear stress around the hole perimeter, with multiple stress concentration points. By comparing the peak values of maximum resolve shear stress around the hole perimeter, it is found that the ideal hole (case 1) and tapered hole (case 5) with variations in the diameter and taper have a maximum resolve shear stress peak difference of only 0.7 MPa. In contrast, picosecond drilling (case 3) with a larger roundness error shows an increase of 20.9 MPa in the maximum resolve shear stress peak compared to the ideal hole (case 1).

**Figure 14:**
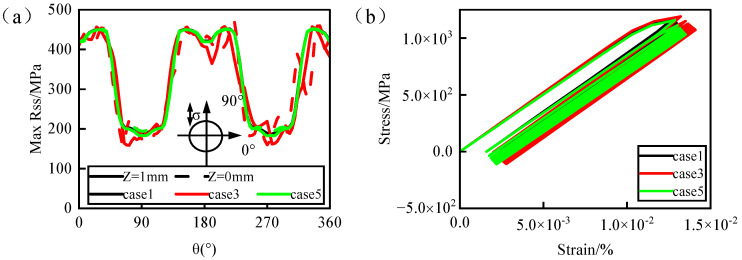
(**a**) The maximum resolve shear stress distribution around the hole; (**b**) ratchet effect at the maximum damage point.

Figure 13 depicts the cam effect curve of the maximum resolve shear stress around the hole perimeter and the maximum damage point under 900 °C isothermal conditions. According to Figure 14a, it can be observed that the distribution of the maximum resolve shear stress around the hole perimeter is consistent for all three cases, aligning closely with the contour maps. However, compared to the ideal hole (case 1) and tapered hole (case 5), picosecond drilling (case 3) exhibits a noticeably irregular and wrinkled cam effect curve of the maximum resolve shear stress around the hole perimeter. This is attributed to the uneven edges and larger roundness error of picosecond drilling (case 3), resulting in multiple stress concentration points within the film-cooling hole. Furthermore, the increased stress concentration phenomena can lead to the initiation of fatigue crack sources, thereby reducing the fatigue life of the film-cooling hole. Comparing the cam effect curves at the maximum damage point in Figure 14b, it is evident that the cam strain curves of the ideal hole (case 1) and tapered hole (case 5) almost overlap, and the cam strains are smaller compared to picosecond drilling (case 3). Moreover, the cam strain in picosecond drilling (case 3) is significantly higher than in the other two from the first cycle, and the subsequent strain accumulation increases gradually. When the strain accumulates to a certain value, material fracture occurs.

Based on the above analysis, in an isothermal environment, a significant roundness error among the geometric accuracy parameters of film-cooling holes notably affects their fatigue behavior, while variations in the diameter and taper have a minimal impact on fatigue behavior.

### 5.2. Fatigue Behavior of Film-Cooling Hole Structures under Real Flow-Field Temperature Conditions

**Figure 15:**
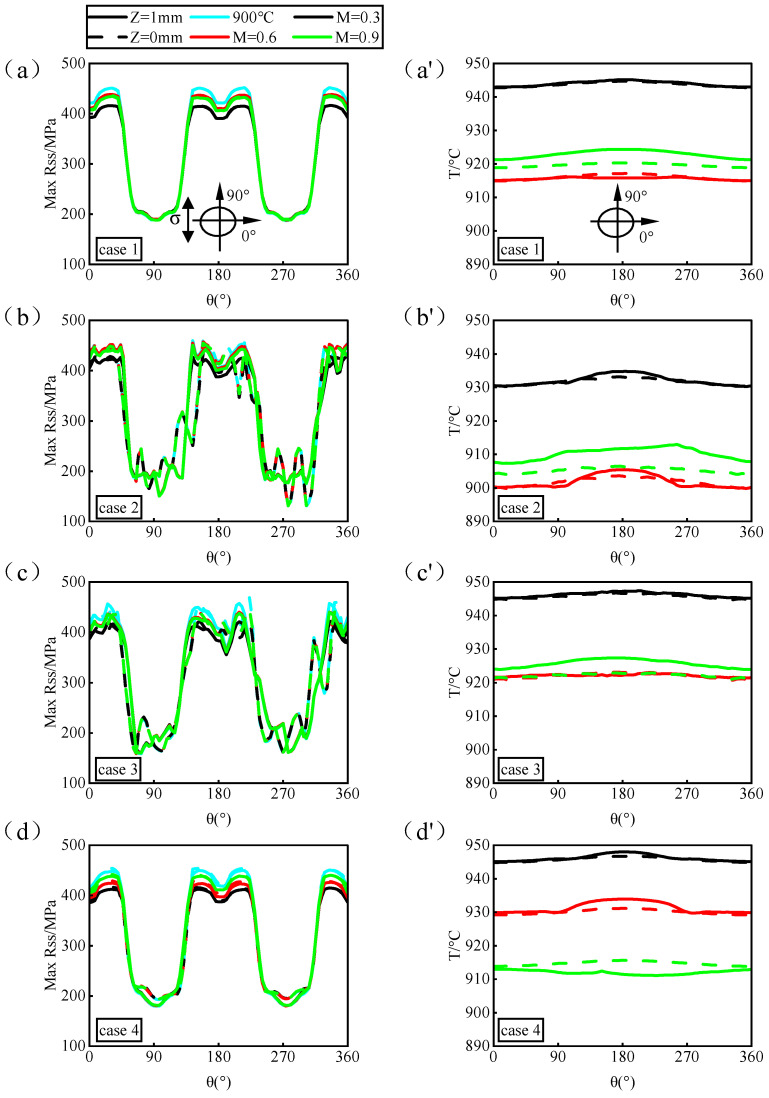
Analysis of maximum resolve shear stress and nodal temperature around the hole. (**a**–**d**) compare the maximum resolve shear stress distribution around the holes for different laser machining processes and the corresponding temperature distribution around the holes is shown in (**a’**–**d’**).

Based on the study of geometric accuracy’s impact on the fatigue behavior of film-cooling holes under isothermal conditions, this section investigates the effect of geometric accuracy on fatigue behavior under real flow-field temperature conditions, leveraging the continuity of temperature fields in the solid domain. Different hole-manufacturing processes were used to generate film-cooling holes in complex temperature fields during film cooling. The analysis was conducted based on the maximum resolve shear stress around the hole perimeter and nodal temperatures. Figure 15 illustrates this analysis: panels a-d depict the distribution of the maximum resolve shear stress around the hole perimeter at different blowing ratios, while panels a’–d’ show nodal temperatures around the hole perimeter at each blowing ratio. It can be observed that the distribution of the maximum resolve shear stress around the hole perimeter for different types of holes remains largely consistent, exhibiting a symmetric pattern with peak values occurring around 0°–60°, 120°–240°, and 300°–360°. However, compared to the ideal hole (case 1) and femtosecond drilling (case 4), both nanosecond drilling (case 2) and picosecond drilling (case 3) show irregular wrinkles in their cam effect curves of the maximum resolve shear stress around the hole perimeter. This consistency with the isothermal field results is due to stress concentration caused by roundness errors. Moreover, compared to picosecond drilling (case 3), nanosecond drilling (case 2) with larger roundness errors exhibits even more fluctuation in its stress curve. This indicates that larger roundness errors lead to more significant stress concentration, resulting in poorer fatigue behavior.

Comparing the distribution of nodal temperatures around the hole perimeter (Figure 15a’–d’), it is evident that changes in the hole type do not significantly affect the temperature field distribution around the hole perimeter. The temperature difference around the hole perimeter generally remains within 5 °C, with overall fluctuations primarily influenced by variations in the hole type and blowing ratio. Combining the analysis of the maximum resolve shear stress around the hole perimeter and nodal temperatures, it is found that changes in the blowing ratio affect the overall temperature field around the hole perimeter, primarily influencing the magnitude of the maximum resolve shear stress peaks. However, the distribution of the stress itself remains unchanged. At lower blowing ratios, higher temperatures around the hole perimeter lead to a decrease in the maximum resolve shear stress peaks, while at higher blowing ratios, lower temperatures around the hole perimeter result in an increase in the maximum resolve shear stress peaks, demonstrating a phenomenon of low stress at high temperatures and high stress at low temperatures. Therefore, the stress values around individual film-cooling holes are temperature-dependent, whereas the correlation between the stress distribution and temperature is minimal, primarily dictated by the geometric structure of the film-cooling holes.

**Figure 16:**
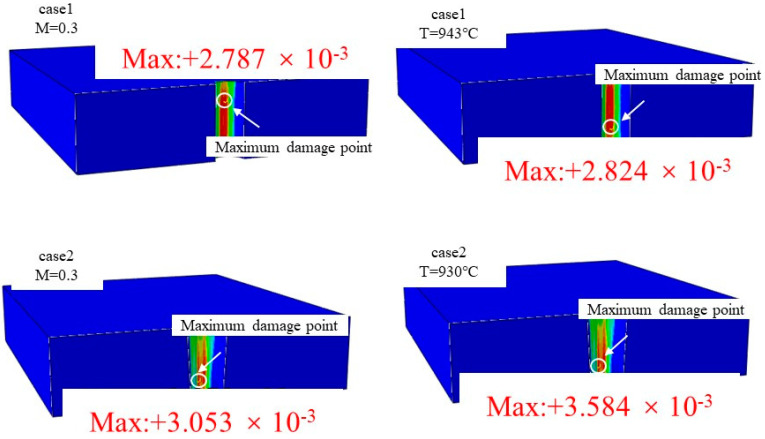
The phenomenon of maximum damage point migration.

Based on the narrow temperature difference observed around individual film-cooling holes, a uniform isothermal field model was constructed to match the actual temperature values around the hole perimeter in the real flow field. This study focused on ideal holes (case 1) and holes formed by nanosecond drilling (case 2), which exhibit significant differences in roundness, taper, and aperture, at a blowing ratio of M = 0.3, considering their impact on fatigue behavior in an isothermal temperature field. As shown in Figure 16, the graph presents the maximum fatigue damage values in complex temperature fields and their corresponding uniform temperature fields. The results indicate that the ideal hole (case 1) shows a shift in the location of maximum fatigue damage points in complex temperature fields, whereas nanosecond drilling (case 2) maintains consistency between the two temperature conditions. This is because, in case 1, being an ideal hole, the impact of hole structural defects on its fatigue behavior remains consistent, with changes in temperature predominantly influencing the extent of damage. On the other hand, the hole formed by nanosecond drilling (case 2) exhibits significant stress concentration due to larger roundness errors in its structural defects. The changes in temperature are not sufficient to cause a shift in the location of maximum fatigue damage points for case 2.

Building on the previous research, it is found that in complex temperature fields, the fatigue damage of individual film-cooling holes is determined by both the hole structural defects caused by different drilling processes and the temperature field around the hole perimeter affecting material performance.

### 5.3. Analysis of Fatigue Behavior of Individual Film-Cooling Holes Manufactured Using Lasers with Different Pulse Widths

**Figure 17:**
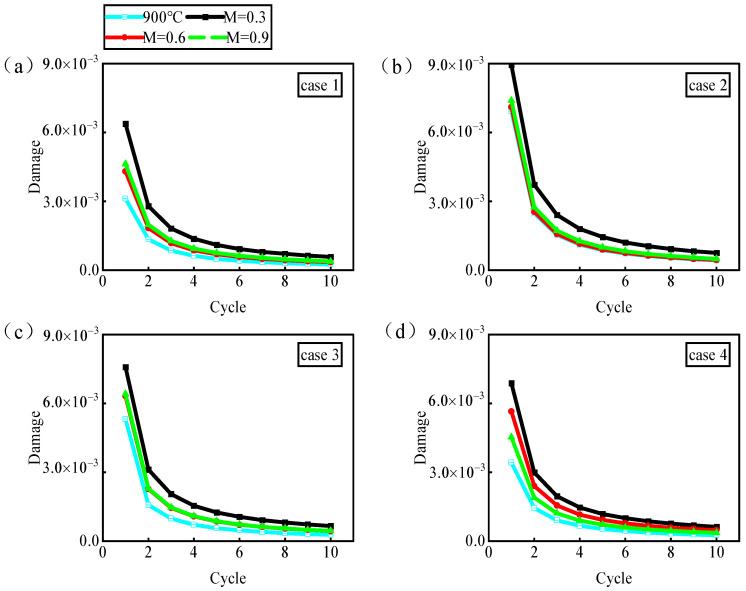
Evolution of fatigue damage. (**a**) Ideal hole. (**b**) Nanosecond. (**c**) Picosecond. (**d**) Femtosecond.

Figure 17 shows the fatigue damage evolution over the first 10 cycles of film-cooling holes manufactured using lasers with different pulse widths. It is observed that under isothermal conditions, compared to the ideal hole (case 1), both nanosecond drilling (case 2) and picosecond drilling (case 3), which have larger roundness errors, exhibit higher fatigue damage values. However, picosecond drilling (case 4), despite having larger taper errors, maintains consistent fatigue damage levels similar to the ideal hole (case 1) and significantly lower than the other two (cases 2 and 3). In an isothermal environment, the geometric parameter of the roundness error strongly influences the fatigue behavior of film-cooling holes, while the diameter and taper have minimal effects on fatigue behavior.

When the blowing ratio changes, resulting in an increase in the temperature around the hole perimeter, the fatigue damage values also gradually increase. Combining this with the distribution of the maximum resolve shear stress around the hole and node temperatures (Figure 15), it can be observed that the fatigue behavior of film-cooling holes exhibits a phenomenon where high temperatures correspond to low stresses and high damage, while low temperatures correspond to high stresses and low damage. This is because the fatigue damage in nickel-based single-crystal DD6 occurs when the maximum resolve shear stress exceeds the critical shear stress. According to Equation (4), the critical shear stress is related to the material’s yield stress. According to the *Chinese High-Temperature Alloy Handbook* [43], the yield stress of the nickel-based alloy DD6 decreases with increasing temperature above 850 °C. Consequently, the critical shear stress also decreases, making it more likely that the lower shear stresses at high temperatures exceed the material’s critical shear stress threshold, thus leading to fatigue damage. Based on this, it can be observed that when the blowing ratio changes, the temperature difference around the hole changes little, resulting in minimal impact on the material’s properties. The fatigue behavior is dominated by the structure of the film-cooling hole, which means that variations in the blowing ratio do not alter the damage distribution around the hole.

**Figure 18:**
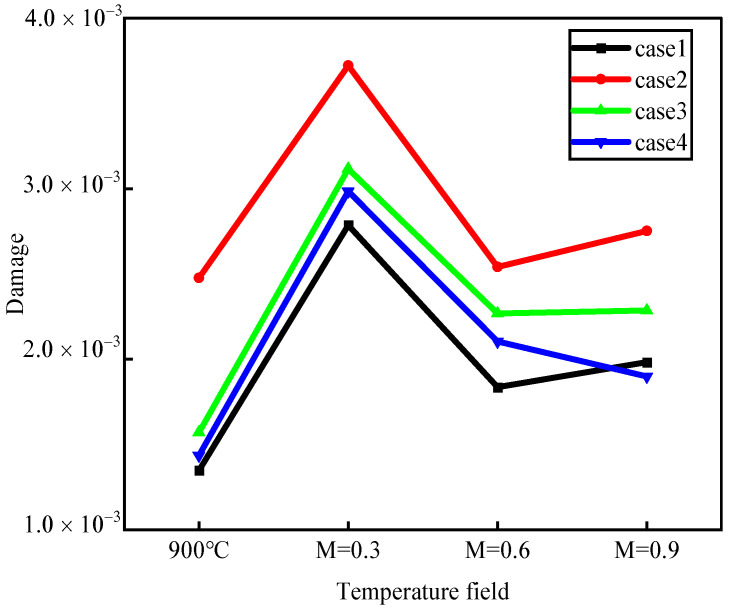
The maximum damage value in the second cycle.

According to Hou et al. [30], adopting the maximum fatigue damage value in the second cycle under temperature gradients can accurately predict the fatigue life of nickel-based single-crystal film-cooling holes, as shown in Figure 18. It can be observed that the fatigue damage values of film-cooling holes manufactured using different drilling processes correlate with the roundness errors of the holes. In this context, the nanosecond drilling process (case 2) consistently exhibits the best cooling performance at any blowing ratio. However, its larger roundness error results in structural defects that predominantly affect the fatigue behavior of the film-cooling holes. Therefore, at any blowing ratio, the fatigue behavior of film-cooling holes manufactured using the nanosecond drilling process (case 2) is the worst, followed by picosecond drilling (case 3) and, finally, femtosecond drilling (case 4). However, at a high blowing ratio (M = 0.9), picosecond laser drilling (case 4) exhibits lower fatigue damage values compared to ideal hole drilling. This is because the smaller roundness error and superior cooling performance of picosecond drilling (case 4) compensate for the damage caused by the smaller roundness error, with the temperature field around the hole dominating the change in material performance. This results in picosecond drilling processes manufacturing film-cooling holes with superior fatigue behavior compared to ideal holes.

**Figure 19:**
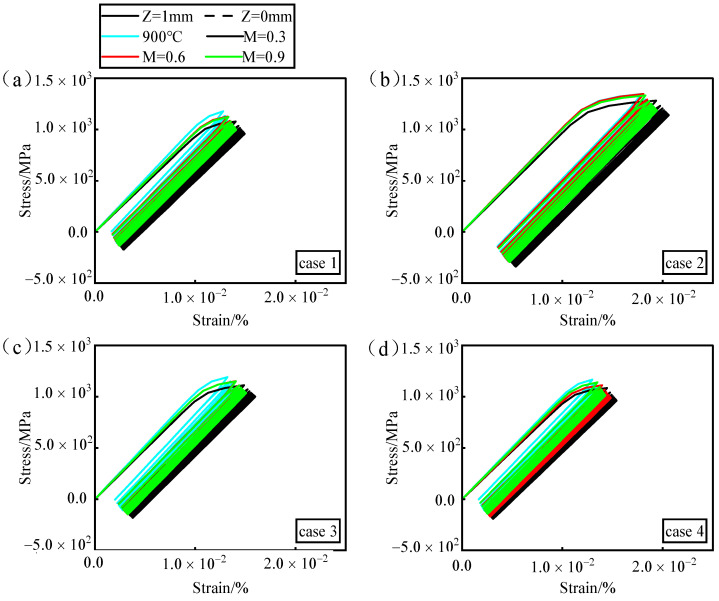
The ratchet effect at the maximum damage point in the second cycle. (**a**) Ideal hole. (**b**) Nanosecond. (**c**) Picosecond. (**d**) Femtosecond.

Based on the temperature gradient described in detail in Section 4.2, the fatigue damage values in the second cycle better correlate with the actual fatigue life. Figure 19 illustrates the ratchet effect at the point of maximum damage in the second cycle. It can be observed that the ratchet strain curve exhibits the highest strain rate in the first cycle, which then rapidly decreases and stabilizes. As ratchet strain continues to accumulate, the material performance progressively deteriorates, leading to fracture after a certain number of cycles.

In different drilling processes, the ratchet strain effects are similar between ideal drilling (case 1) and picosecond laser drilling (case 4). However, nanosecond drilling (case 2) and picosecond drilling (case 3) exhibit significantly higher ratchet strain levels compared to ideal drilling (case 1), with a greater accumulation of strain over subsequent cycles. Comparing nanosecond drilling (case 2) and picosecond drilling (case 3), nanosecond drilling (case 2) exhibits significantly higher levels of ratchet strain than picosecond drilling (case 3). Within the same drilling process, as the blowing ratio varies, higher temperatures around the hole perimeter correspond to greater ratchet strain levels and the increased accumulation of ratchet strain. This indicates that material fatigue behavior deteriorates more rapidly in high-temperature regions compared to low-temperature regions. When the accumulation reaches a certain threshold, material failure initiates from the high-temperature areas.

The aforementioned research aligns with the earlier discussion on film-cooling hole damage evolution. Fatigue damage in film-cooling holes is influenced jointly by structural defects caused by different drilling processes and changes in material performance due to temperatures around the hole. Structural factors have a greater influence on this interaction.

## 6. Conclusions

This research investigates the complex temperature distribution and fatigue behavior of single film-cooling holes manufactured by lasers with different pulse widths in a real flow field. The aerodynamic and heat transfer characteristics of film-cooling holes manufactured using lasers with different pulse widths were analyzed through laser drilling experiments, conjugate heat transfer simulations, and crystal plasticity finite element methods. The results showed the following:

(1) The diameter and taper of film-cooling holes significantly influence their aerodynamic and heat transfer characteristics, while roundness has no impact. However, roundness errors notably affect the fatigue behavior of film-cooling holes and exhibit a negative correlation. Conversely, the diameter and taper hardly alter their fatigue behavior. The influence of the structure on fatigue behavior is greater than the influence of temperature. Therefore, in design, it is a priority to optimize the structure while maximizing cooling performance as much as possible.
(2) In a real flow field, a single film-cooling hole exhibits second-order thermal distribution characteristics. The first order is dominated by film cooling, evident in the temperature gradient along the downstream hot wall surface of the hole. The second order is controlled by heat conduction between the gas and solid, manifested as the lowest temperature around the hole perimeter, with temperature gradients extending outward and increasing. Based on the conclusions of this study, further equivalent research using real flow-field temperature tests can be conducted. Establishing a quantitative conversion relationship between equivalent boundaries and real boundaries can be used to guide mechanical testing.
(3) Changes in the blowing ratio do not affect the temperature or stress distribution around the hole perimeter; they only alter the temperature peak values. An increase in temperature peaks leads to a decrease in stress peak values. According to crystal plasticity theory, DD6 exhibits a positive correlation between fatigue damage and temperature above 850 °C. This phenomenon results in the occurrence of low damage with high stress at low temperatures and high damage with low stress at high temperatures. This has practical application value for material selection and thermal management strategies in high-temperature environments.
(4) The fatigue damage of a single film-cooling hole is jointly determined by structural defects in the hole and changes in material performance due to temperatures around the hole, with structural factors exerting a greater influence. Nanosecond laser processing exhibits significant machining errors, resulting in oversized apertures, roundness issues, and excessive taper. While its cooling effect is optimal, the roundness error notably decreases fatigue behavior. Picosecond laser drilling follows next in preference. On the other hand, femtosecond laser processing, due to its smaller roundness error, demonstrates superior fatigue behavior.

## Figures and Tables

**Table 1:** The elemental composition of the DD6 superalloy (wt %).

Element	Co	W	Ta	Al	Cr	Re	Mo	Ni
Content	8.5~9.5	7.0~9.0	6.0~8.5	5.2~6.2	3.8~4.8	1.6~2.4	1.5~2.5	Bal

**Table 2:** Geometric accuracy of film-cooling holes manufactured by lasers with different pulse widths.

Number	Laser Type	Upper Hole Diameter (µm)	Lower Hole Diameter (µm)	Upper Hole Diameter Error	Lower Hole Diameter Error	Upper Hole Roundness (µm)	Lower Hole Roundness (µm)	Taper (°)
Case 1	Ideal hole	400.0	400.0	0	0	0	0	0
Case 2	Nanosecond	539.2	448.6	34.80%	12.15%	0.0318	0.0357	7.41
Case 3	Picosecond	396.4	385.2	−0.90%	−3.70%	0.0246	0.0346	0.92
Case 4	Femtosecond	450.2	382.8	12.50%	−4.30%	0.0047	0.0131	5.50

**Table 3:** Material constants of the dynamic strengthening law.

C	a	d	Q	R	α	n	$Y_{st}$
6.0	150.0	4.5 × 10^–7^	85.0	8.31	1.6 × 10^–7^	4.275	5.0

**Table 4:** DD6 fatigue damage model parameters at different temperatures.

Temperature (°C)	*E* (MPa)	*v*	*G* (MPa)	$\tau_{0}\left(MPa\right)$	$g_{0}^{\alpha }\left(MPa\right)$	$n_{fat}$
25	131.50	0.344	155.07	382.6	366.9	0.61
760	105.50	0.377	115.43	376.0	408.0	0.65
850	91.46	0.383	105.85	357.2	410.5	0.71
980	80.50	0.390	85.60	240.0	275.0	0.37
1070	69.50	0.399	72.50	200.0	206.5	0.24

**Table 5:** Heat transfer parameters of DD6 high-temperature alloy.

Temperature (K)	Heat Transfer Coefficient (W (m·K))	Specific Heat Capacity (J (kg·K))
773.15	15.35	496
973.15	20.20	566
1173.15	24.55	635
1273.15	26.80	669
1373.15	28.95	704
1473.15	33.20	773

**Table 6:** Geometric accuracy parameters of film-cooling holes (isothermal field (T = 900 °C)).

Number	Laser Type	Upper Hole Diameter (µm)	Lower Hole Diameter (µm)	Upper Hole Roundness (µm)	Lower Hole Roundness (µm)	Taper (°)
Case 1	Ideal hole	400.0	400.0	0	0	0
Case 3	Picosecond	385.2	380.9	24.6	34.6	0.35
Case 5	Tapered hole	450.0	400.0	0	0	4.10

## Data Availability

Data will be made available on request.
